# Molecular Imaging of Tau Protein: New Insights and Future Directions

**DOI:** 10.3389/fnmol.2020.586169

**Published:** 2020-12-15

**Authors:** Rocco Pizzarelli, Natalia Pediconi, Silvia Di Angelantonio

**Affiliations:** ^1^Center for Life Nanoscience, Istituto Italiano di Tecnologia (IIT), Rome, Italy; ^2^Department of Physiology and Pharmacology, Sapienza University, Rome, Italy

**Keywords:** tau, super-resolution imaging, neurodegeneration, BODIPY, near-infrared fluorescent probes, Alzheimer’s disease, tauopathies

## Abstract

Tau is a microtubule-associated protein (MAPT) that is highly expressed in neurons and implicated in several cellular processes. Tau misfolding and self-aggregation give rise to proteinaceous deposits known as neuro-fibrillary tangles. Tau tangles play a key role in the genesis of a group of diseases commonly referred to as tauopathies; notably, these aggregates start to form decades before any clinical symptoms manifest. Advanced imaging methodologies have clarified important structural and functional aspects of tau and could have a role as diagnostic tools in clinical research. In the present review, recent progresses in tau imaging will be discussed. We will focus mainly on super-resolution imaging methods and the development of near-infrared fluorescent probes.

## Introduction

Tauopathy is a general term referring to a group of disorders characterized by the accumulation of misfolded tau protein. Highly heterogeneous from a clinical perspective, tauopathies can be further divided into primary tauopathies (where tau is the leading cause of neurodegeneration) and secondary tauopathies (where a tauopathy is associated with other pathologies). Progressive supranuclear palsy (PSP), Pick’s disease (PiD), and fronto-temporal dementia (FTD) are examples of primary tauopathies while, Alzheimer’s disease (AD), Lewy’s body disorder (LBD), and Down’s syndrome (DS) belong to the second group (Arendt et al., 2016; Saha and Sen, 2019). Tau is a microtubule-associated protein (MAPT) highly expressed in neurons where it takes part in several physiological functions vital to the cell. Since the discovery of tau as a major component of neurofibrillary tangles (NFT; Grundke-Iqbal et al., 1986; Kosik et al., 1986; Wood et al., 1986), researchers have focused their attention to the study of its features. Indeed, a better understanding of tau functions could contribute to shed light on the basic mechanisms involved in the genesis of tauopathies. Tau aggregates start to form in the brain 10–15 years before the manifestation of any clinical symptoms, thus presenting an appealing possibility to diagnose and monitor tauopathies by using tau aggregates as an early biomarker. This has strongly encouraged the development of biomedical advanced methodologies for its visualization. Positron emission tomography (PET) is the golden standard for imaging-based diagnosis of neurodegenerative disorders. However, several research groups are trying to develop alternative strategies. In addition, studies examining tau biology through super-resolution microscopy (SRM) are beginning to become available. In the present article, we would like to provide a short overview about some of the recent imaging methodologies adopted to visualize tau protein, discussing their strengths, limitations, and future developments.

## Tau Super-Resolution Imaging

A major drawback of light microscopy is represented by the resolution limit imposed by light diffraction that does not allow objects located at a distance <200 nm apart to be separated (Sigal et al., 2018). Light microscopy renders a detailed examination of proteins and organelles involved in cellular processes, an arduous task. However, ground-breaking progress in the field of microscopy has allowed the development of new imaging techniques commonly termed super-resolution microscopy (SRM). These techniques provide a spatial resolution as low as 20–50 nm (Sahl et al., 2017); currently, their application spans different areas of life sciences research. One of the recent super-resolutive techniques is Stimulated Emission Depletion Microscopy (STED). The main advantage offered by this method relies on the fact that it allows features of fluoresce microscopy to be combined, while bypassing the diffraction limit (Vicidomini et al., 2018). STED microscopy has been used to investigate the distribution of physiological tau protein at mossy-fiber synapse in the CA3 hippocampal region of the adult mouse brain (Kubo et al., 2019). In this work, tau has been described as nonuniformly distributed and rather sparsely localized at the level of axonal microtubules, with a distance of ~200 nm. According to the authors, this organization suggests that physiological tau does not saturate axonal microtubule (MT) binding. The same imaging technique has been adopted to visualize tau with a resolution of 77 nm in *postmortem* AD human brain slices (Benda et al., 2016). Immunofluorescence with the phospho-tau (p-tau) primary antibody 12E8 followed by STED imaging, revealed that the protein is organized mainly in filaments characterized by a smooth ribbon-like structure. However, the presence of puncta 70–80 nm wide has been reported as well. Colocalization of tau with both stable and dynamic microtubules and with actin filaments has been observed at the level of fixed growth cones in cultured cortical neurons (Biswas and Kalil, 2018). Reducing tau levels with small hairpin RNA (shRNA) did not affect cone morphology but was responsible for a reduction of axon outgrowth and growth cone turning. Human excitatory cortical neurons derived from patients with FTD have been used to investigate the effect of the autosomal-dominant *MAPT*P301L and IVS10+ 16 tau mutations on the protein function. In FTD-MAPT neurons, hyperphosphorylated tau mislocalize to cell bodies and dendrites; moreover, three-dimensional (3D) STED experiments, revealed that in FTD neurons, an aberrant microtubular organization was responsible for the deformation of the nuclear lamina (Paonessa et al., 2019).

First introduced in 2006 (Betzig et al., 2006), stochastic optical reconstruction microscopy (STORM) is a powerful method for protein localization. STORM achieves an image resolution below 20 nm and is based on the random activation of fluorophores with photoactive properties followed by image reconstruction. Using this technique, exogenous tau fibrils clustering at the neuronal membrane has been investigated by performing imaging experiments on cultured hippocampal neurons after application of preformed tau fibrils labeled with ATTO-647 dye. While the density of exogenous tau fibrils binding to neurons increased over time, the radius of clusters was not affected (Shrivastava et al., 2019). STORM has been combined with neuropathological immunohistochemistry methods to image ptau in the prefrontal, parietal, and temporal cortex of AD patients with a resolution <50 nm (Codron et al., 2020). Tau aggregates appeared organized in filamentous structures at the axonal level while at the soma, they presented a honeycombed structure most likely arising from the presence of proteins and organelles. These results confirm and extend those obtained by Benda et al. (2016). Last, single-molecule localization microscopy (SMLM) has been used to investigate the tau organization at the nanoscale level in both physiological and pathological conditions (Gyparaki et al., 2020).

**Figure 1:**
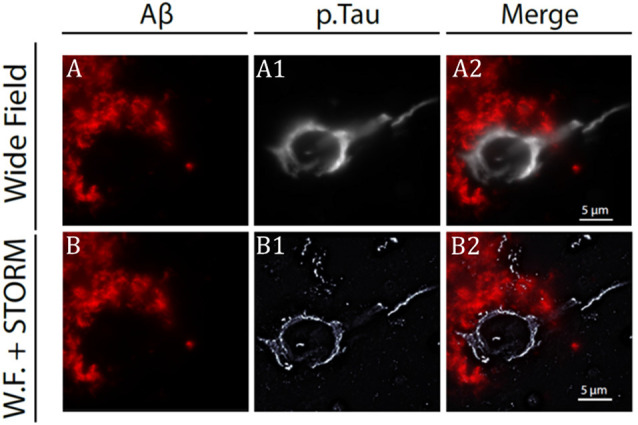
Stochastic optical reconstruction microscopy (STORM) image of beta-amyloid plaque and neurofibrillary tangles (NFT) in Alzheimer’s disease (AD) patient brain slice. **(A)** Confocal microscopy image of beta-amyloid aggregates (in red) and neuro-fibrillary tangles **(A1)** from an AD patient brain specimen. Super-imposed images are shown in panel **(A2)**. Same senile plaque as in panel **(A)**, imaged by combining fluorescence microscopy for beta-amyloid aggregate **(B)** with STORM microscopy for NFT **(B1)**. Super-imposed images are shown in panel **(B2)**. Note the difference in resolution between image in panels **(A2)** and **(B2)**. Modified from Codron et al. (2020) under the Creative Commons CC-BY-NC-ND.

Although few in number, works reviewed above considered different aspects of tau protein biology, demonstrating how, super-resolution imaging methods represent a powerful approach to unravel the less well-understood aspects of tau structure and function both in physiological and pathological conditions. Single molecule tracking and cryo-electron microscopy studies have indicated tau-MT interaction as weak and dynamic (Janning et al., 2014; Kellogg et al., 2018; see Barbier et al., 2019 for review); in line with this, recent SRM studies seem to favor a nonuniform rather than diffuse tau distribution along the microtubules. A possible explanation for this could be that, previous studies mostly performed by conventional light microscopy approaches were unable to solve structures with distances <200 nm (see Figure 1 for a comparison between the resolution level of confocal and STORM microscopies). SRM is particularly useful in that they allow to monitor protein dynamics *in situ*, both in fixed and living specimens, opening the way for the study of the initial steps of protein aggregation and their dynamics over time. Works examining these aspects at subcellular level are starting to become available (Schierle et al., 2016; Lu et al., 2020); however, some technical aspects such as long-term imaging, fluorophore bleaching, and specimen thickness are still major obstacles that need to be overcome (see “Conclusion and Future Directions” section).

## Pet Probes

A definitive diagnosis for neurodegenerative disorders can be solely made from *postmortem* immunohistochemical examination of brain samples (Delacourte, 1998). Nevertheless, analysis of biomarkers in the cerebrospinal fluid (CSF) combined with high-resolution imaging techniques and clinical examination is an elective method for the diagnosis of neurodegenerative disorders in living patients. Imaging tau in AD patients could be very important from a clinical point of view as it has been suggested that NFT density seems to show a better correlation with neurodegeneration and cognitive impairments compared with Aβ plaques (Dickson et al., 1992). This aspect is of interest as it could serve for disease prediction and staging and for the evaluation of the effectiveness of pharmacological treatments. Therefore, beyond the diagnostic value, tau visualization on a temporal and spatial scale could also assume prognostic significance. PET uses isotope-conjugated tracers able to bind to molecules of interest ideally with high affinity and specificity in order to visualize them. PET has been successfully used in order to visualize β-amyloid plaques. Since the pioneering article published by Klunk et al. (2004), a plethora of tracers have been proposed. More recently, the approach already undertaken for the visualization of beta-amyloid aggregates has been extended to NFT. However, the peculiar structural organization and localization of this protein constitute a major challenge for the development of reliable radiotracers (Villemagne and Okamura, 2016; Leuzy et al., 2019). First, in NFT, tau can be present under different isoforms and/or posttranslational modifications. In addition, NFT are located mainly intracellularly; therefore, selective tracers need to cross the plasma membrane. Among the first generation of tau PET tracers, there are [^18^F] THK5351, [^18^F]THK5317, [^18^F]AV1451 (also known as flortaucipir), and [^11^C]PBB3. These compounds represented a “proof of concept” in the process of tau PET imaging and their properties have extensively been examined (Leuzy et al., 2019). A rigorous and final validation step for PET tracer requires verification of the correspondence between *antemortem* PET images with *postmortem* immunohistochemical analysis in the same patient. Despite its usefulness, implementing this paradigm is not easy and, to date, very few studies are available. Correlation analysis has been performed by Harada et al. (2018) on an autopsy-confirmed AD patient (81 years old). The man had a history of progressive memory impairment and cognitive decline and underwent PET scanning with the [^18^F]THK5351 tracer before death (Harada et al., 2018). The authors reported that *in vivo* cortical [^18^F]THK5351 retention correlated not only with *postmortem* tau immunohistochemistry but also with monoamine oxidase-B (MAO-B) in the whole brain; in addition, MAO-B correlated with glial fibrillary acid protein (GFAP) a marker for astrocytes, and therefore they concluded that [^18^F]THK5351 PET could be useful to evaluate tau-associated neuroinflammatory states in AD rather than tau pathology. PET scanning using the tracer [^18^F]AV-1451 was performed on a 76-year-old female patient carrying the MAPT R406W mutation. This mutation leads to the presence of tau aggregates similar to those found in AD. Clinically, the patient presented a long history of cognitive deficits and behavioral abnormalities. *Postmortem* immunohistochemical staining for hyperphosphorylated tau protein showed a strong positive correlation between *in vivo* [^18^F]AV-1451 retention and the density of tau aggregates (Smith et al., 2016). PET scans with the same tracer have been performed on three male subjects (age 77, 68, and 56 years old, respectively) affected by non-Alzheimer tauopathy (two PSP and one *MAPT* P301L mutation carrier). In this study, no significant correlation was reported between *in vivo* PET and autoptic examination of tau deposits; therefore, the authors conclude that [^18^F]-AV-1451 may not be suited to detect tau aggregates in these non-AD tauopathies (Marquié et al., 2017). Lastly, [^18^F] flortaucipir *antemortem* PET imaging scans have been compared with *postmortem* immunohistochemical identification of tau pathology using a larger cohort compared with previous studies (Fleisher et al., 2020). As the authors suggest, some limitations were present in the study (patients older than normally selected for clinical trials and tracer retention in subcortical structures as a potential off-target effect); however, they conclude that the overall results obtained support the ability of flortaucipir to bind to NFT and its role in AD diagnosis.

Works reviewed here clearly demonstrate the difficulties in obtaining selective ligands for tau. A major concern is represented by off-target effects. Nonspecific binding could be due to the presence of β-sheet motives, a common feature of protein aggregates other than NFT (such as TDP-43, α-synuclein, et cetera). Another big obstacle is represented by the heterogeneous structural organization of tau aggregates in AD and non-AD-related tauopathies. The need for dyes with better selectivity, affinity, and specificity is opening the door for the development of a second generation of tau tracers (see Kolb and André, 2017). Tau structure has recently been described at atomic level (Fitzpatrick et al., 2017; Kellogg et al., 2018). It is likely that in the future, growing knowledge in this field will lead to the development of better tau radiotracers to be used for clinical purposes.

## Near-Infrared Probes

**Figure 2:**
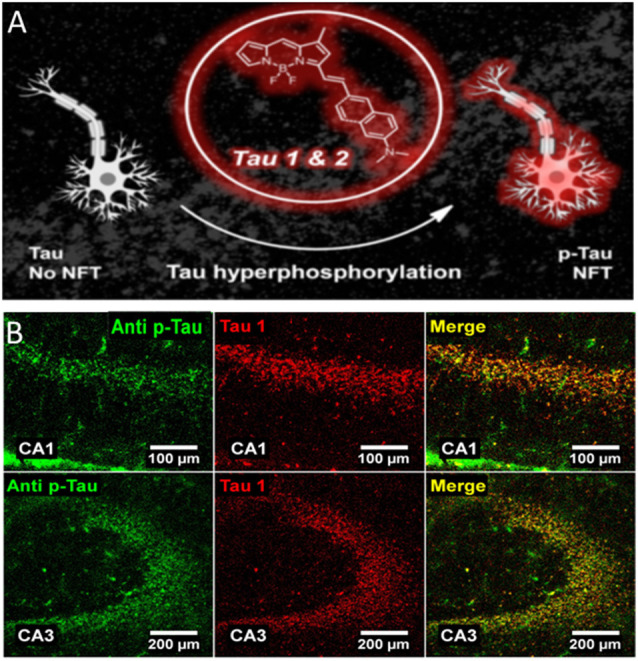
4,4-difluoro-4-bora3a,4a-diaza-s-indacene (BODIPY)-based probe binds to neurofibrillary tangles in a mouse model of AD. **(A)** Near-infrared probes (NIR) Tau 1&2 bind to NFT structures. **(B)** Immunostaining showing the colocalization between pTau (green signal) and Tau1 (red signal) in the CA1 (upper panels) and CA3 (lower panels) hippocampal region of a mouse treated with okadaic acid. Colocalization of the two signals is shown. Adapted with permission from Verwilst et al. (2017). Copyright 2017 American Chemical Society.

Several research groups are actively involved in developing affordable probes/methodologies that could be potentially used for the diagnosis of tau-based neurodegenerative disorders. A plethora of approaches using different chemical strategies have been proposed (Verwilst et al., 2018). In the present paragraph, we will examine near-infrared probes (NIR) exclusively as these seem to be good candidate probes for future *in vivo* application. NIR are a class of compounds characterized by an emission wavelength ranging from ~650–900 nm of the electromagnetic spectrum. Optical probes with an emission wavelength falling in this range facilitate photon penetration into tissue, reducing the effects of autofluorescence and light scattering (Hilderbrand and Weissleder, 2010). With respect to the development of PET probes, the availability of NIR probes for tau aggregates is limited compared with that for the Aβ amyloid plaques. Compounds based on boron dipyrrin platform (4,4-difluoro-4-bora3a,4a-diaza-s-indacene, also known as BODIPY) have been receiving additional interest for their potential biomedical applications. These compounds are endowed with interesting spectroscopic and photodynamic features that can easily be customized through relatively simple chemical reactions thus making them suitable for several applications (Durantini et al., 2018). Based on molecular docking observations, the synthesis of two BODIPY-based probes selective for tau (Tau1 and Tau2) has been described (Verwilst et al., 2017). These probes presented a large Stokes shift, emission at ~650 nm and high selectivity for tau. Immunofluorescence experiments performed following okadaic acid (OA) injection in adult mice, further confirmed that Tau1 is capable of labeling hyperphosphorylated tau as costaining with the antibody AT-180 revealed good overlap between the two signals (Figure 2). A similar result was also observed using the 3xTg AD mouse model. Beta-amyloid and tau aggregates are known to be labeled by curcumin, a natural pigment derived from the rizoma *Curcuma longa*. Following structural modification of CRANAD-2, a NIR curcumin-based probe selective for beta-amyloid (Ran et al., 2009), a tracer for tau aggregates has been proposed. The derivative **1c** was characterized by a significant change in fluorescence upon binding to tau fibrils, but fluorescence emission wavelength was slightly off from the NIR range (~620 nm). *In vitro* experiments confirmed its ability to label intracellular tau aggregates; however, no experiments on mouse models of tauopathies nor on *postmortem* human AD tissues have been performed (Park et al., 2015). Two more probes (**3g** and **3h**) with better fluorescence emission upon tau aggregates binding (*λ*em = 650 nm) have been proposed (Seo et al., 2016); in this case, probe selectivity was tested both *in vitro* and on AD human brain slices. Immunofluorescence staining revealed good merging between signals from the **3g** or **3h** probe and the signal given by an antibody against ptau. Lastly, the compound **2e** is characterized by a high specificity for tau over beta-amyloid, a very interesting fluorescence emission (*λ*em = 660 nm) and a large Stokes shift (110 nm). When incubated with AD human brain slices, the probe **2e** displayed a staining pattern that resembled that which was obtained with an antibody against ptau epitopes (Ser202/Thr205; Park et al., 2017). A different strategy using small tau antibody fragments (scFV) conjugated to a near-infrared fluorescent probe has been reported (Krishnaswamy et al., 2014, 2018). scFv antibodies have a smaller size compared with full-length antibodies and offer the possibility to target specific tau epitopes and are more selective over β-sheet dyes. Following NIR-labeled scFv235 injection into two mouse models of tauopathy, *in vivo* imaging system (IVIS) experiments allowed a strong and stable signal from the brain of both JNPL3 and htau/PS1 mice to be detected. Very little signal was observed in control animals. Moreover, older mice from both genotypes showed higher signals compared with younger mice further suggesting the specificity of the scFv 235 antibody (Krishnaswamy et al., 2014).

## Conclusions and Future Directions

Advanced imaging methodologies are providing valuable information about tau structure, interaction with proteins and organelles, localization, and mechanisms of self-aggregation. Far from being definitive, these techniques are still under active development as several limitations need to be overcome. A critical step for improvement is represented by protein labeling with fluorescent probes (Wang et al., 2019). Fluorophore-conjugated antibodies or synthetic compounds have been used for this purpose; however, in some cases, their size could represent an obstacle (Sahl et al., 2017). Also, it is reasonable that the fluorophore may cause steric hindrance thus altering protein trafficking or delicate mechanisms of protein–protein interaction. Last, the incomplete labeling could lead to spurious results; this represents a very critical point for the study of *in vitro* aggregation of misfolded proteins in particular (Cosentino et al., 2019). It is likely that in the future a refinement of labeling methods for SRM combined with label-free microscopy (Schierle et al., 2016) could allow these problems to be overcome. The development of selective probes for diagnostic purposes is a very active field of research. NIR-based probes have been proposed as a valuable approach for the labeling of tau aggregates. Works reviewed here have indicated reasonably high specificity of these compounds both *in vitro* and *in vivo*. Tau aggregates have recently been described in the retina of AD patients (Schön et al., 2012; den Haan et al., 2018; Grimaldi et al., 2019). It has therefore been proposed that retinal imaging of tau aggregates could have a role for AD diagnosis (Ngolab et al., 2019). Although further investigations are needed, NIR probes represent a valuable tool for this purpose. However, aspects such as low quantum yield and short emission wavelength must be taken in account. Also, the solubility of these compounds and the need for a pharmacophore could represent a clear obstacle to overcome for further human biomedical application. New imaging methodologies combined with information about tau structure will have a profound impact on both clinical and basic tau research.

## Author Contributions

All authors reviewed and discussed relevant literature, wrote sections, and approved the final version of the manuscript.

## Conflict of Interest

The authors declare that the research was conducted in the absence of any commercial or financial relationships that could be construed as a potential conflict of interest.

## Abbreviations

AD: Alzheimer’s disease
CSF: cerebrospinal fluid
DS: Down’s syndrome
FTD: frontotemporal dementia
GFAP: glial fibrillary acid protein
GFP: green fluorescent protein
IVIS: *in vivo* imaging system
LBD: Lewy body disorder
MAO-B: monoamine oxidase-B
MAPT: microtubule-associated protein tau
NFT: neurofibrillary tangles
NIR: near infrared
OA: okadaic acid
PiD: Pick’s disease
PSP: progressive supranuclear palsy
PET: positron emission tomography
ScFv: single chain antibody fragment
shRNA: small-hairpin RNA
SMLM: single-molecule localization microscopy
SRM: super-resolution microscopy
STED: stimulated-depletion microscopy
STORM: stochastic optical reconstruction microscopy.
